# Supplementary figures and images for: Acquired Hemophilia A after SARS-CoV-2 Infection: A Case Report and an Updated Systematic Review
Source: Biomedicines. 2023 Aug 28;11(9):2400. doi: 10.3390/biomedicines11092400 (PMC10526109; doi:10.3390/biomedicines11092400)

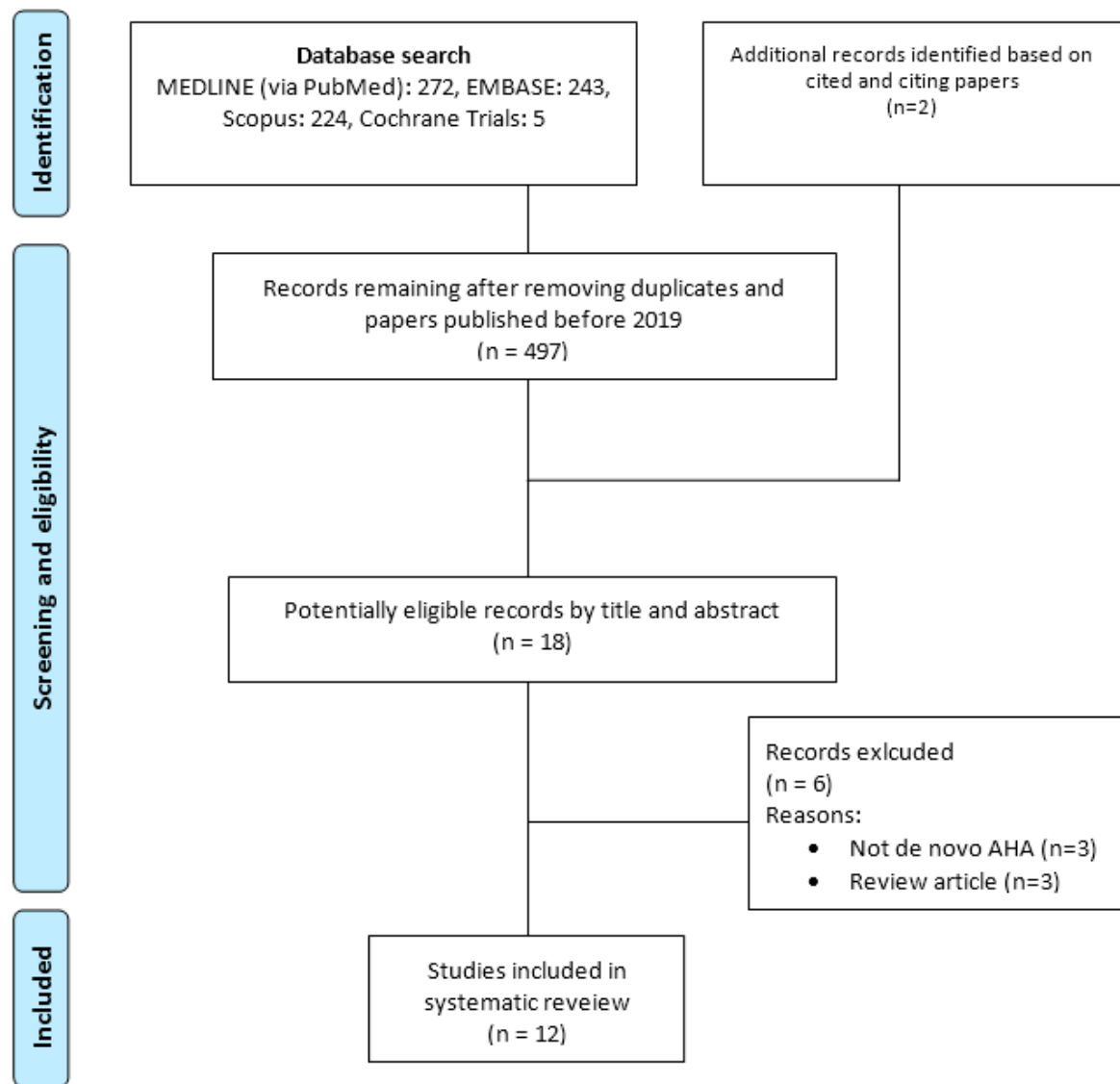

Figure S1. Flow chart of search and selection.

Supplement: Supplementary file 1 [file biomedicines-11-02400-s001.zip › biomedicines-2514267-supplementary.pdf]
